# Vasectomy and male sexual dysfunction risk: A systematic review and meta-analysis

**DOI:** 10.1097/MD.0000000000022149

**Published:** 2020-09-11

**Authors:** Fang Yang, Liang Dong, Xiaojin Zhang, Junjun Li, Kun Tan, Yulin Li, Xujun Yu

**Affiliations:** a Department of Andrology, The Reproductive & Women-Children Hospital, Chengdu University of Traditional Chinese Medicine, Chengdu, Sichuan, P. R. China; b Chengdu University of Traditional Chinese Medicine, Chengdu, Sichuan, P. R. China.

**Keywords:** male sexual dysfunction, meta-analysis, protocol, systematic review, vasectomy

## Abstract

**Background:**

Unintended pregnancy is popular all over the world, accounting for 40% to 50% of all pregnancies. The condition not only exerts pressure on the relationship of couples and severely impacts the quality of life, but also imposes a heavy burden on the health of women and child. Recently, more than 220 million couples have chosen to be sterilized to obtain contraception, 47.3% of married couples select sterilization, of which vasectomy accounts for 17.1%. Vasectomy is currently the most convenient and effective method of male contraception. We will perform the systematic review and meta-analysis to assess the correlation between vasectomy and male sex dysfunction and provide evidence-based evidence for the couple

**Methods:**

The electronic databases of MEDLINE, PubMed, Web of Science, EMBASE, Clinicaltrials.org., China National Knowledge Infrastructure Database (CNKI), Wan fang Database, China Biology Medicine Database (CBM), VIP Science Technology Periodical Database, Chinese Clinical Trial Registry, and Cochrane Library will be retrieved before November 20, 2021. We will search English literature and Chinese literature with proper Medical Subject Heading or text key words. RevMan 5.3 and Stata 14.0 will be used for Systematic review and Meta-analysis. This protocol reported in accordance with the Preferred Reporting Items for Systematic Reviews and Meta-Analyses Protocols (PRISMA-P) statement, and we will report the systematic review by following the PRISMA statement.

**Conclusion and dissemination:**

The aim of this study was to evaluate the effect of vasectomy on the sexual function of patients after operation. The results will be published in a public issue journal to provide evidence-based medical evidence for urologists and andrologists to make clinical decisions.

**Registration information:**

INPLASY202080014.

## 1 Introduction

Unintended pregnancy is popular all over the world, accounting for 40% to 50% of all pregnancies.^[1]^ Whereas, several women will choose to obtain abortions suffering the unintended pregnancy. The condition not only exerts pressure on the relationship of couples and severely impacts the quality of life (QoL), but also imposes a heavy burden on the health of women and child, such as damaging the mental health of women,^[2]^ affecting subsequent pregnancy,^[3]^ and even inducing child maltreatment.^[4]^ Therefore, selecting appropriate contraception is necessary. Meanwhile, the method of contraception is increasing due to the changes in sexual activities and reproductive intentions.^[5]^ More than 220 million couples have chosen to be sterilized to obtain contraception, 47.3% of married couples select sterilization, of which tubal ligation accounts for 30.2% and vasectomy accounts for 17.1%. Vasectomy, as the only form of permanent male sterilization, the success rate of it is 99.7%, with a lower incidence of complications of 1% to 2%.^[6]^ This method is only applicable to men who do not want to have children in the future.

Vasectomy, which must be done voluntarily, is currently the most convenient and effective method of male contraception, mainly resulting from its simplicity, low invasiveness, efficiency, and relatively low incidence of complications. Studies have shown that 96% of men will show severe oligospermia or azoospermia after 12 weeks of vasectomy,^[7]^ and common complications such as hematoma, infection, chronic scrotal pain, and pain at the surgical site and pain and pressure occur within the abdomen.^[6]^ A massive number of studies have shown that vasectomy did not affect male sexual function, and interestingly, had a positive effect on the sexual function of the female partners.^[8]^ A key factor may be the anxiety of women about unwanted pregnancies was reduced, and women's libido improved somewhat, and they were more willing to cooperate with men in sex.^[9]^

In the past, although several researches^[10–13]^ have indicated that vasectomy is an effective and safe option to get contraception, they still lack more evidence to support this viewpoint. Hence, we will perform the systematic review and meta-analysis to assess the correlation between vasectomy and male sex dysfunction and provide evidence-based evidence for the couple who would like to get the vasectomy to reduce the worry, anxiety, and depression of them.

## 2 Review objectives

The purpose of this study is to research the effect of vasectomy on male sexual function. The evaluation of sexual function is mainly conducted by the International erectile function questionnaire-5 (IIEF-5) and general information questionnaire, collecting the data of sexual desire, erectile function, frequency, and duration of sexual intercourse were collected. This study will provide more evidence to clarify the relationship between vasectomy and erectile dysfunction.

## 3 Methods

This is a systematic review, with a meta-analysis if necessary. The data and results used in this paper are almost from published studies, and there are no ethical issues, so the approval of the ethics committee is not required.

### 3.1 Protocol and registration

This study is registered on PROSPERO. Registration number: CRD42020158346. This protocol refers to the statement of Preferred Reporting Items for Systematic Reviews and Meta-Analyses Protocols (PRISMA-P).^[14,15]^ And we will report the systematic review by the PRISMA statement strictly.

### 3.2 Data source

#### 3.2.1 Electronic search database and approach

The electronic databases of MEDLINE, PubMed, Web of Science, EMBASE, Clinicaltrials.org., China National Knowledge Infrastructure Database (CNKI), Wan fang Database, China Biology Medicine Database (CBM), VIP Science Technology Periodical Database, Chinese Clinical Trial Registry, and Cochrane Library will be retrieved before November 20, 2021.

Grey literature will be searched in Open Grey. Related randomized controlled trials (RCTs) will be collected and selected before November 20, 2021. The searching work will be done in December 2021 and updated before the systematic review has completed.

Medical Subject Heading or text key words “Vasectomy” or “Vasectomies” or “Vas Ligation” AND “Dysfunction, Erectile” or “Male Sexual Impotence” or “Impotence, Male Sexual” or “Sexual Impotence, Male” or “Male Impotence” or “Impotence, Male” or “Impotence” will be used. The search strategy is adjusted to fit the different databases. The Chinese form of the above terms will be used in Chinese search. A specific search example for PubMed is summarized in Table 1.

**Table 1:**
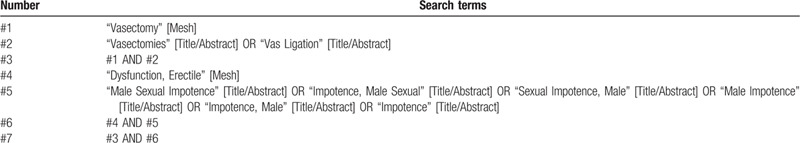
PubMed search strategies.

#### 3.2.2 Other sources of search

Grey literature will be retrieved through Open Grey. The acquisition of full-text documents requires library interlibrary loans and Google academic. A manual review of references in published articles will be conducted to identify other relevant studies, and references detected from existing system reviews and meta-analyses will also be retrieved.

### 3.3 Included and excluded criteria

#### 3.3.1 Study design

Randomized, double-blind, placebo-controlled trials would be identified the best. Because this is surgical treatment, so it is difficult to achieve random, controlled for surgical treatment. As long as the criteria of PRISMA are met, relevant clinical trials can be systematically reviewed and meta-analysis can be conducted if necessary. Therefore, some other suitable research types can be included.

#### 3.3.2 Participants

**Included population**

- The mental health male patient who underwent a vasectomy.
- Before operation, the sexual function of patients was normal.
- It would be better if the sexual function of the patients was evaluated authoritatively before the operation.
- Age >18 years.

**Excluded population**

- Other contraceptive methods are used.
- Patients with some diseases that can cause sexual dysfunction.
- Patients with psychological diseases, and other related diseases or conditions.

#### 3.3.3 Interventions and controls

*Treatment group:* All patients received vasectomy, regardless of the operation mode. There are no other contraceptive methods and no other factors causing sexual dysfunction, including psychological factors.

*Control group:* A placebo with the same appearance as the treatment group or normal healthy people.

#### 3.3.4 Outcomes

**Primary outcome indicator:**

(1) IIEF.
(2) Ejaculation function (premature ejaculation, delayed ejaculation, non-ejaculation, ejaculation pain, etc).
(3) Sexual desire.

**Secondary outcome indicators:**

(1) Postoperative pain scores.
(2) QoL.

### 3.4 Selection of studies and data extraction

Document management will be conducted by Endnote X9 software. The software will be used to filter duplicate documents first, and then delete duplicate documents by reading titles, abstracts, and other relevant information.

According to the inclusion criteria and exclusion criteria, the literature will be further screened. In this process, the controversial literature will be screened after obtaining the full text. Further detailed screening and data extraction of the documents will be carried out simultaneously by 2 professionally trained reviewers.

**Figure 1:**
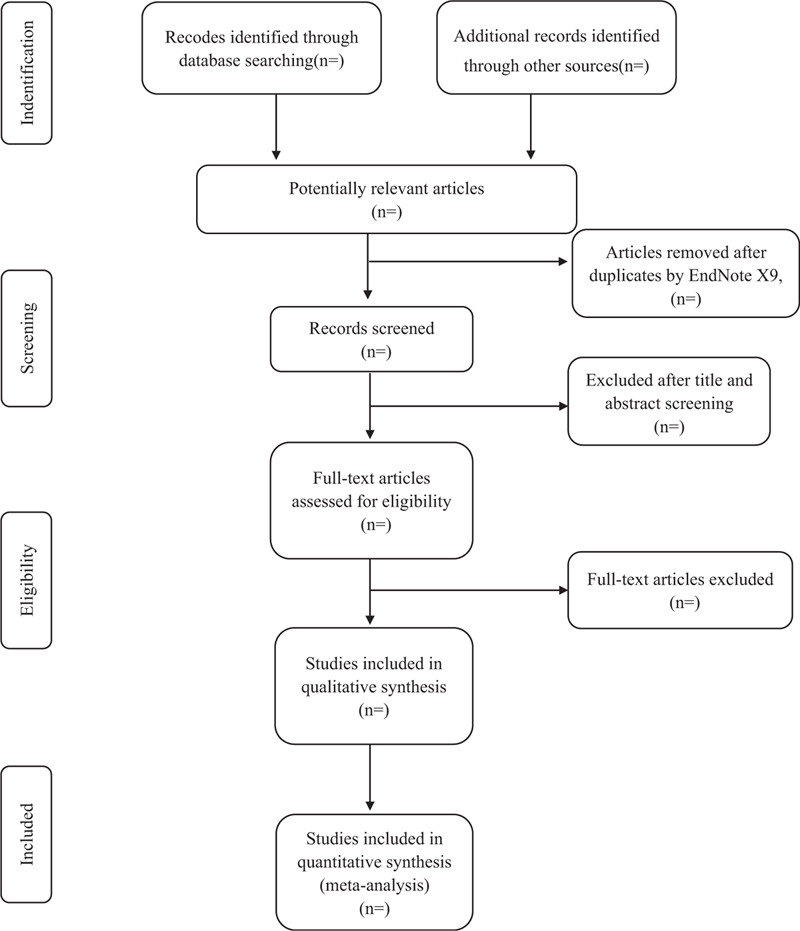
The PRISMA literature screening flow chart.

Then, the articles that meet the inclusion criteria are full-text read and rescreened. If 2 or more articles have repeated or staged research results, only the articles with the largest sample size, the most complete intervention, and follow-up time are included. When the review team cannot confirm the repeated studies, the original study author will be contacted for judgment. The flow chart of literature screening is shown in Fig. 1.

After completing the screening and removing duplicates, all articles that compared different types of vasectomy or RCTs (including conference papers that can be obtained by contacting the author for the original research details) will be included. Then, after discussion within review group, a unified data extraction form (an excel spreadsheet) will be produced. Extracted information will include characteristics and methodology of included studies, participant characteristics, details of interventions and control measures, data information for outcome indicators, and other data including information for assessment of the risk of bias. Before the formal data extraction, 2 review authors will independently conduct data extraction exercises. All differences will be discussed and resolved with the third reviewer.

### 3.5 Risk of bias assessment

Selection bias, performance bias, detection bias, attrition bias, reporting bias, and other bias will be assessed based on the Cochrane Collaboration Network Risk Assessment Tool. Two review authors will independently evaluate and cross check the risk of bias. Discrepancies between review authors on the risk of bias will be resolved through discussion with a third review author.

Assessment items include random sequence generation, allocation concealment, blinding of participants and personnel, blinding of outcome assessment, incomplete outcome data, selective reporting, and other bias.^[16]^ Each item of bias situation includes low risk, unclear, and high risk. As we cannot determine the authenticity of blindness, the outcome indicators of the systematic review are relatively objective, so we define the generation of random sequence, allocation concealment, and incomplete data as key domains of risk of bias evaluation. The risk of bias assessment chart of inclusion studies will be produced by using Review Manager 5.3 software.

### 3.6 Data analysis and synthesis

Descriptive analysis or narrative synthesis will be performed when there are clinical heterogeneity among the studies or when the data cannot be synthesized or results data cannot be extracted. When included trials are clinically homogeneous and the data are similar and synthesizable, a meta-analysis will be performed. Dichotomous variable will be pooled as risk ratio (RR) and 95% confidence intervals. Continuous variable will be pooled as mean difference (MD) and 95% confidence intervals. We will use Cochran's Q statistic and *I*^2^ statistic to test heterogeneity. *P* < .10 is heterogeneous, or *I*^2^ > 50% is significant heterogeneity. A fixed effect model (Mantel--Haenzel method for RR and Inverse Variance for MD) will be used for *I*^2^ < 50%. A random effects model (D-L method) will be used when the heterogeneity is still significant after sensitivity analysis and subgroup analysis. A *P* < .05 of *z* test will be considered statistically significant. The meta-analysis will be generated by Review Manager 5.3 software and displayed as a forest plot, while a funnel plot will be generated to assess the risk of bias.

### 3.7 Subgroup analysis

If the data are sufficient and there is heterogeneity between studies, we will perform a subgroup analysis:

(1) different kind of vasectomy;
(2) different measurement methods;
(3) different comorbidity;
(4) demographic characteristics of the patients: age, marital and family status, ethnicity;
(5) follow-up time.

### 3.8 Sensitivity analysis

Sensitivity analysis will be used to test the reliability and stability of the meta-analysis results, and to assess the source of heterogeneity. This can be done by excluding trials with a high risk of bias or eliminating each study individually. The meta-analysis will then be performed again and the results compared with the previous meta-analysis.

### 3.9 Publication bias

Publication bias will be measured by using a funnel plot (by Review Manager 5.3 software), Begg test, and Egger test (by Stata software 14.0).

## 4 Discussion

Many patients are concerned about the association between vasectomy and sexual function and worry that the quality of their sexual lives might be affected after surgery. Fortunately, most studies have thus far shown that vasectomy does not affect sexual function or even improve it. Studies carried out in the 1980s showed that vasectomy had a positive psychological effect on patients, improving their sexual lives, harmony between couples, and sexual desire and increasing the frequency of sexual intercourse.^[17–19]^ Several studies undertaken in recent years have also confirmed that men after undergoing a vasectomy experience markedly improved erectile function, orgasms, and sexual satisfaction and feel safer and more confident in their sexual lives after surgery.^[8,11,12,13,20]^ Their female partners reported marked improvements in terms of sexual arousal, satisfaction, and orgasm, as well as lubrication and libido. In addition, Guo et al^[9]^ showed that men who had undergone a vasectomy experienced more instances of sexual contact per month than men who had not undergone a vasectomy.

Performance anxiety can be caused by several factors, such as stress and fear, which result in the release of the adrenaline and noradrenaline, with consequent contraction of the smooth muscles in the corpus cavernosum that results in detumescence and thus the inability to maintain an erection long enough to complete sexual intercourse. During recent decades, few reports have shown that men are more likely to develop symptoms of depression and anxiety after vasectomy.^[21,22]^

Whether vasectomy affects the sexual function is attracting more attention of the patients who would like to suffering vasectomy. Hence, with the aim to reply the confusion, we will assess the correlation between vasectomy and sexual function.

## Author contributions

Conceptualization: Fang Yang. Data curation: Liang Dong, Junjun Li. Formal analysis: Xiaojin Zhang, Kun Tan. Methodology: Yulin Li. Project administration: Fang Yang. Software: Liang Dong Supervision: Junjun Li Validation: Fang Yang. Writing – original draft: Xujun Yu Writing – review & editing: Xujun Yu, Fang Yang.

**Conceptualization:** Fang Yang.

**Data curation:** Liang Dong, Junjun Li.

**Formal analysis:** Xiaojin Zhang, Kun Tan.

**Methodology:** Yulin Li.

**Project administration:** Fang Yang.

**Software:** Liang Dong.

**Supervision:** Junjun Li.

**Validation:** Fang Yang.

**Writing – original draft:** Xujun Yu.

**Writing – review & editing:** Fang Yang, Xujun Yu.
